# Epidemiology and Risk Factors for Rheumatoid Arthritis Development

**DOI:** 10.31138/mjr.301223.eaf

**Published:** 2023-12-30

**Authors:** Aliki I. Venetsanopoulou, Yannis Alamanos, Paraskevi V. Voulgari, Alexandros A. Drosos

**Affiliations:** 1 Department of Rheumatology, School of Health Sciences, Faculty of Medicine, University of Ioannina, Ioannina, Greece,; 2 Institute of Epidemiology, Preventive Medicine and Public Health, Corfu, Greece

**Keywords:** rheumatoid arthritis, incidence, prevalence, environmental factors, genetic factors

## Abstract

Rheumatoid arthritis (RA) is a prevalent chronic inflammatory arthritis worldwide, significantly impacting patients and population health. The disease affects women primarily, with a female-to-male ratio of three to one. Its pathogenesis is multifactorial, including genetic and environmental risk factors. Epidemiological studies highlight the link between the environment and genetic susceptibility to RA. The so-called shared epitope is the most significant risk factor that seems to act synergetic with other environmental factors in the disease occurrence. In addition, recent findings suggest a potential role of new substantial environmental factors, such as the observed pollution of the planet’s natural resources, on the susceptibility and progression of the disease. This review summarises the most decisive evidence on epidemiology and genetic, environmental, and lifestyle risk factors for RA. It shows that studying genetic and environmental factors in correlation could lead to prevention strategies that may impact the natural history of the disease.

## DEFINING RA

Rheumatoid arthritis (RA) is a chronic, inflammatory, autoimmune disease that impacts approximately 0,5-1% of the adult population and presents two- to three-fold more frequently in women than in men. Although the disease is heterogeneous, with various systemic manifestations, the hallmark of the advanced disease is the persistent inflammation of the synovium, which usually involves peripheral joints in a symmetrical distribution.^1,2^ RA has a multifactorial aetiology and can be considered the final result of an interplay between epigenetic processes and environmental factors that act in genetically predisposed individuals.^3,4^ In the serum of RA patients, several autoantibodies have been identified, including rheumatoid factor (RF), anti-citrullinated protein antibodies (ACPA), and anti-carbamylated protein antibodies.^5^ The role of these autoantibodies is critical as they may form immune complexes in the joint that contribute to the inflammatory processes that lead to articular cartilage damage. Depending on the presence or absence of RF and ACPA in RA patients’ serum, two forms of the disease have been established: Seropositive RA, which represents the most common form and is characterised by the presence of RF and or ACPA antibodies, while seronegative RA is determined by the absence of both RF and ACPA.^6^

In recent years, early diagnosis and treatment initiation with disease-modifying antirheumatic drugs (DMARDs) in a treat-to-target strategy have led to rapid disease remission and improved outcomes for RA patients.^7–9^ RA has no diagnostic criteria, but classification criteria exist based on clinical and lab features such as symptom duration, joint involvement, serology, and acute-phase reactant levels. These criteria define patient groups for studies on disease progression and treatment response.^10–12^

Understanding the epidemiology and risk factors associated with RA is crucial in light of the changing disease patterns, emerging treatment options, and the importance of personalised healthcare approaches for different disease subsets. Factors such as environmental conditions, genetics, epigenetics, comorbidities, and patient-reported outcomes should be considered while analysing the epidemiology of RA.

## METHODS

We conducted a narrative review using the Medline and PubMed search engines to find articles in English published without time limitations on publication dates. We used the following keywords: “rheumatoid arthritis,” “epidemiology,” “prevalence,” “incidence,” “mortality,” “risk factor,” “genetic,” “epigenetic,” “hormonal,” “environment,” “infectious agents,” “socio-economic status,” “air pollution,” “occupational dust,” “smoking,” “alcohol,” “vitamin D deficiency,” and “diet”.

The articles that discussed the epidemiology, environmental, and genetic risk factors related to RA were selected after a screening process carried out by the authors. We did not perform any meta-analyses as our objective was to address various questions in a descriptive manner rather than to obtain summarized estimations for one or two risk factors.

### Epidemiological Aspects

Epidemiologic principles have been used to describe the distribution of RA in the population and to examine possible risk factors for disease occurrence and progression.^13^ The different sets of classification criteria that may have been used to define the disease in epidemiological studies may have influenced the results. Other important parameters include the reference population and the sample size. At the same time, a lack of standardisation for the characteristics responsible for the differences in comparison may lead researchers to inconsistent conclusions.^14^ Appropriate attention to methodological issues can resolve these issues.^15^ Following that, most researchers use the 1987 American College of Rheumatology (ACR) criteria for the definition of RA and include in studies a sufficient sample size of individuals over 16 years old, while most recent studies report the age-adjusted prevalence and incidence rates. However, a potential inherent bias in population studies remains challenging to control.

#### Prevalence of RA

Most studies on RA estimate periodic prevalence, considering the flare-and-remitting nature of the disease. Still, potential biases may result from different socioeconomic, demographic, and healthcare conditions in each country; This should be kept in mind, especially when comparing Western with Third World populations, where significantly more than 40% of the population may be under 25 years of age.^16^ Moreover, when interpreting studies results, someone should be cautious about whether age-standardized prevalence rates or crude rates are used. As many prevalence studies have been conducted decades apart, differences in prevalence rates may also reflect temporal variations rather than differences between populations.^17^

Overall, RA’s global prevalence has been estimated to range from 0.24 to 1%, although rates vary by country and geographical region.^18^ The disease seems less common in Africa and Asia than in the United States of America and Europe.^19^ At a population level, the highest prevalence worldwide of self-reported disease has been observed in Australia (2%), according to the data of a National Health Survey during 2014–2015.^20^ At a community level, Native American populations, such as Pima and Chippewa tribes,^21,22^ present the highest rates that have ever been reported (5.3% and 6.8%, respectively). On the contrary, reports from rural populations in South Africa (0.0026%)^23^ and Nigeria (0%)^24^ indicate a very low prevalence or even absence of the disease.^25^

#### Incidence of RA

The number of published community-based incidence studies is relatively low compared to those referring to RA prevalence. These studies are problematic to complete as extended periods of population follow-up are needed, while it is challenging for those who rely on medical records to find adequate medical data resources.^17^ Nevertheless, the existing data demonstrate a temporal variability in the disease’s incidence with different trends depending on the region. In some populations, RA incidence initially increased in the late 60s and has since decreased.^26^ This variation was predominately observed since the early 90s in white populations in Europe^27,28^ and the United States of America,^29,30^ including the indigenous Pima Indians.31 The decline in the disease’s incidence among whites was mainly observed among women,^29^ while the idea of a declining RA incidence was supported by the coincidental decrease in the disease’s prevalence and the rate of positive RF in young RA patients.^32^ On the contrary, RA incidence seems to increase in other regions like Africa.^33^ Yet, when interpreting data from developing countries, someone should keep in mind that the lower RA incidence observed in those countries compared to northern European and American ones may in fact reflect differences in the age distribution between the studying populations.

#### Mortality

Higher standardised mortality ratios (SMRs) for all-cause mortality, up to 1.5–1.6, have been reported in RA patients than in the general population,^34–36^ which in some cases have been related to the disease severity.^37^ Moreover, the hazard ratio for death has been reported to increase even in under-treatment patients, possibly associated with longer disease duration.^38^ The primary causes of death in RA patients are thought to be cardiovascular diseases, respiratory diseases, and infections.^34^ An interesting meta-analysis of data from the last 50 years showed a decrease in mortality in RA patients, which nevertheless remained higher than in the general population.^39^ Other studies have shown the same trend over time,^40–42^ although results from some others differ.^43^ The conflicting results may derive from methodological issues as to different types of cohorts and different follow-up times but also may relate to the changing treatment over the last two decades.

However, there is a tendency to underreport RA on death certificates, especially in older patients with a more significant number of comorbidities, and thus decrease the likelihood that RA would be included when completing the death certificate.^44^ Long-term clinical cohorts with a large sample of RA patients should be performed to overcome these discrepancies using more recent mortality data.

### Risk factors for RA

**Figure 1.**
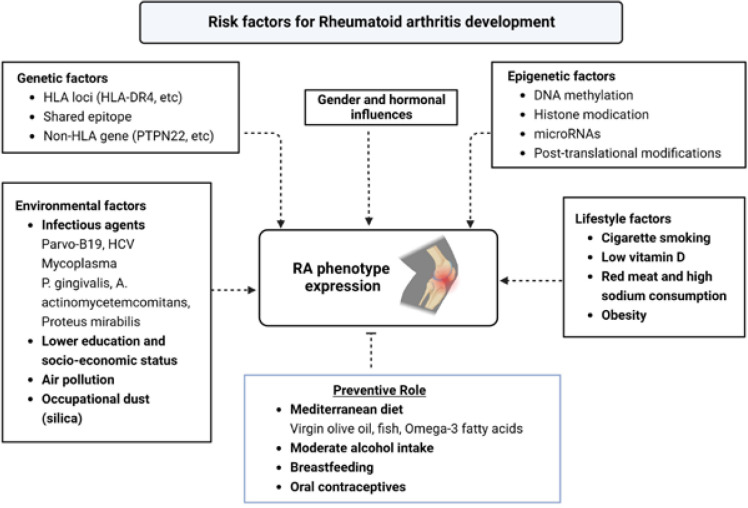
Risk factors associated with RA development and phenotype expression.

For the last decades, much knowledge has been gained on the predisposing factors to RA. Multiple environmental factors, along with genetic ones, contribute to the disease’s development. There are inherited risk factors passed down from parent to child through genes. Many other factors are related to environmental and lifestyle choices seemingly controllable by individuals, such as tobacco smoking (**Figure 1**). There is also support for the protective role of other factors, including certain dietary foods. However, we cannot predict RA, and understanding the risk factors and their interplay is essential for a better assessment and management of the disease.

## THE GENETIC AND EPIGENETIC IMPACT ON RA

RA presents a robust genetic background. Twin studies have revealed that the disease’s heritability, independently from environmental factors, reaches almost 60% in cases of ACPA-positive patients, while estimates for the seronegative disease are lower.^45–47^ The disease has long been associated with human leukocyte antigen (HLA) genes. Among the genetic risk factors, the shared epitope (SE), a sequence of a five-amino acid motif encoded by HLA-DRB1 alleles, is the most significant one.^48^ The SE is associated with an increased RA susceptibility, an earlier disease onset, more severe cartilage erosions, and the presence of ACPAs.^49^ Distinct genetic patterns between the seropositive and seronegative subsets of the disease have been defined according to ACPA status.^50^

Genome-wide association studies (GWAS) using single nucleotide polymorphisms (SNPs) have also revealed disease-associated variants in the population that may cumulatively influence RA development. Polymorphic variants of the tyrosine phosphatase non-receptor type 22 (PTPN22) gene, the Cytotoxic T-Lymphocyte Associated Protein 4 (CTLA4), the signal Transducer and Activator of Transcription 4 (STAT4), and variants of the genes that encode enzymes peptidylarginine deiminases (PADI)-2 and PADI-4 have been associated with susceptibility to RA.^51–53^ Still, differences in the genetic factors related to RA susceptibility have been identified between different populations in Europe, Asia, and Africa.^54,55^

Epigenetic changes, which affect the expression of many genes, have also been proposed to influence RA pathogenesis. So far, several epigenetic changes have been reported, including DNA methylation, histone modifications, and non-coding RNAs, also called microRNAs (miRNAs).^56^ Post-translational modifications (PTM), including glycosylation, citrullination, and carbamylation, are associated with the disease onset in predisposed individuals.^57^

## GENDER AND HORMONAL INFLUENCE ON RA

Studies indicate that women are approximately twoto-three times more likely to present RA than men.^58^ They also have a more aggressive disease course with greater activity and disability.^59^ The spontaneous amelioration during pregnancy and the definite increase in RA prevalence in women around menopause have led investigations to hypothesise that hormonal factors play a role in the development of the disease. Although the published data are conflicting, declining oestrogen and/or progesterone levels in post-partum and menopause seem to augment the risk and severity of RA.^60^ The peak age of RA in women around menopause is accompanied by a sharp increase in serum follicle-stimulating hormone (FSH) levels. In some studies, high FSH levels are a possible risk factor for RA and are positively associated with disease activity.^61^ On the other hand, breastfeeding is associated with a lower risk of developing RA, independently of the duration of breastfeeding.^62^

Exposure to Oral contraceptives (OC) is related to a reduced risk of RA development and improvement of the established disease activity and severity.^63,64^ OC use has a lower protective effect on the risk of RA with a change in OC composition.^65^ On the contrary, hormone replacement therapy is associated with a small but significant increase in the risk of seropositive RA.^66^

## THE ROLE OF THE ENVIRONMENT IN RA

Environmental factors like climate change, pollution, and disease-causing microbes can lead to chronic diseases like RA. Poor lifestyle choices such as smoking and poor nutrition can also contribute to chronic diseases. The underlying mechanism of this effect is poorly understood, and more research is necessary to develop effective prevention strategies and promote healthier lifestyles.

### Exposure to infectious agents

Different pathogens may trigger autoimmunity through molecular mimicry, epitope spreading, bystander activation, and subdominant cryptic antigens.^67^ Studies on RA after viral exposure are few and have poor quality. Parvo B19 and hepatitis C virus previous infection relate to RA occurrence, while Cytomegalovirus and hepatitis B virus infections are not associated with the disease. Infection with Epstein-Barr virus (EBV) has been suggested to contribute to the pathogenesis of RA. However, a recent meta-analysis did not show that prior infection with EBV predisposes RA development.^68^ Interestingly Chikungunya virus is associated with persistent inflammatory arthritis with RA-like arthritis clinical features.^69^

Regarding COVID-19 caused by the SARS-CoV-2 virus, the data are limited. Although some RA-reported cases follow a COVID-19 infection, it is uncertain if they present a coincidence rather than a connection of the virus infection to the disease occurrence.^70,71^ Nevertheless, there is evidence that the SARS-CoV-2 virus may disturb immunological tolerance, and further studies are needed. Clinical and animal model studies have also suggested that microbiomes, such as Aggregatibacter actinomycetemcomitans, Porphyromonas gingivalis (P. gingivalis), Proteus mirabilis, and mycoplasma contribute to the etiopathogenesis of RA. Among those, P. gingivalis is thought to be a significant infectious agent in individuals with periodontal disease, which has been associated with an increased risk of developing RA.^72–74^

The association between infection during the first year of life and the risk of RA at age 16 years or later has been shown to have a stronger association for seronegative RA, although these associations were based on small numbers.^75,76^ Still, other possible perinatal factors related to foetal growth may be critical to the disease occurrence, and more research is needed before any conclusions can be made.

### Socio-economic status

Socioeconomic status is a hierarchical social classification associated with the income, educational level, occupational class, social class, and the origin of each individual. These parameters present conditional interactions with different outcomes in health and disease.^76^ Few reports exist in the literature showing that lower educational level, occupation, income, and living in rural areas may influence the disease activity or even act as a possible risk factor for the disease occurrence.^78,79^ Conversely, having a higher socioeconomic status has been associated with a lower incidence of RA.^80^ Interestingly, lower socioeconomic status is correlated to other factors related to the disease, including smoking, obesity, poor nutrition, and a higher overall frequency of chronic disease. On the contrary, higher education and income are related to an early disease diagnosis.^81^

### Air pollution

Environmental air pollution mainly comes from energy use and production. Pollutants with the most substantial evidence for public health concerns include particulate matter (PM), ozone (O3), carbon monoxide, nitrogen dioxide (NO2), sulphur dioxide (SO2), and lead.^82^ The consequences of air pollution on humans depend on the type of pollutant, the length and level of exposure, the cumulative impact in case of multiple pollutants, and each individual’s health issues. The pathophysiology linking environmental air pollution and the development of autoimmune diseases implicates the lung as an autoimmunity initiation organ; Airway damage induced by inhaled exposure may lead to systemic inflammation, increased oxidative stress, and epigenetic modifications, all of which are crucial for the development of chronic diseases such as RA.^83^ Long-term exposure to ambient pollution has been associated with a higher risk of auto-immune diseases.^84^ Living near air pollution emitters has been associated with a higher likelihood of developing RA^.85-87^ Of different air pollutants measures, exposure to fine PM2.5 appears to be most closely linked to ACPA titers.^88^ Some studies have also observed a striking association between air pollution and RA disease severity and flares, where exposure to high levels of air pollutants was associated with increased C-reactive protein (CRP) levels and a higher risk of a disease flare.^89^

Still, there is no strong epidemiological evidence that may relate one specific air pollution particle to the occurrence of RA. Air pollution is a complex mixture, and it is not easy to use adequate methodologies correlating meteorological variables with RA. Focusing on specific components may decrease the likelihood of demonstrating any significant association.

### Occupational dust (silica)

In the year 2000 and only in the EU, more than 3.2 million workers were exposed to silica dust in their professions, including workers at miners, constructions, and ceramic, quarry, and pottery industries.^90^ Studies have linked inhaled silica dust to silicosis, sarcoidosis, and chronic obstructive pulmonary disease.^91–93^ The epidemiological studies that investigated a possible association between inhaled silica and the occurrence of RA, indeed revealed an increased risk for the disease.^94,95^ The hypothesis behind this relation includes evidence of significantly higher levels of Interleukin (IL) 1α, IL-1β, IL-2, IL-4, IL-6, IL-10, and tumor necrosis factor-alpha (TNF-α) in ceramic workers, in addition to an impaired antioxidant/oxidant status suggestive of a triggered immune system and inflammatory response to the exposure to silica.^96^ This association in some studies seems to increase with the number of years exposed to silica.^97^

## LIFESTYLE FACTORS

### Exposure to tobacco smoke

Cigarettes are one of the top environmental considerations in RA patients. Smoking has a significant impact on immune responses. It increases the body’s oxidative stress, interferes in apoptosis, provokes proinflammatory processes, and causes citrullination, and epigenetic changes, such as DNA methylation.^98^ It is evident that past and current cigarette smoking (CS) are related to the development of RA, in particular seropositive RA, where intensity and duration are directly related to the risk, with a prolonged increased risk even after cessation.^99,100^ A gene-environment interaction exists between smoking and the HLA-DRB1 SE genotype. The relative risk of seropositive RA has been reported to be remarkably high in smokers carrying single or double SE alleles.^101^ CS not only increases the risk of seropositive RA but also might influence the severity and clinical expression of the disease.^102,103^ CS can also impact treatment response to DMARDs and affect future joint damage.^104^ On the contrary, behavioral modification with sustained SC could delay or prevent seropositive disease.^105^

Compared to smoking cigarettes, vaping involves heating an “e-juice” in a battery-powered device to create a vapor. The vapor contains nicotine and ultrafine particles that can worsen or trigger chronic diseases when inhaled into the lungs.^106^ Currently, no data is available regarding the risk of RA associated with vaping.

### Alcohol consumption

Low to moderate consumption of alcohol is associated with a reduced risk of RA in a dose-dependent, time-dependent and sex-dependent manner.^107,108^ The relation is more pronounced for ACPA-positive RA.^109^ A meta-analysis based on eight prospective studies suggested that the impact of alcohol consumption on RA risk might follow a J-shaped curve, and people with low-to-moderate alcohol consumption may have a lower RA risk.^110^ Moreover, a synergistic effect between alcohol and smoking has been reported, where the positive association between smoking and RA incidence was reduced when increasing alcohol consumption.^111^

Alcohol consumption is also associated with lower disease activity and self-reported health assessment in RA.^112^ A three-way interaction between alcohol, smoking, and HLA-DRB1-SE has been reported regarding the risk for ACPA-positive RA.^113^ These findings emphasize the need to investigate interactions between several environmental and genetic factors in order to understand the disease occurrence better.

### Vitamin D deficiency

Vitamin D, as a fat-soluble vitamin and a steroid pre-hormone, is believed to possess an immune-modulatory effect.^114^ Research suggests that Vitamin D intake has been inversely associated with RA risk.^115,116^ Vitamin D supplementation for five years, with or without omega-3 fatty acids, has been shown to reduce the incidence of an autoimmune disease, including a 40% reduction in RA incidence.^117^ Some studies also report a negative association between serum vitamin D and disease activity. In order to overcome unmeasured confounding, which is a major limitation of observational studies, researchers have started to use Mendelian randomisation, with genetic variants as instrumental variables for all modifiable risk factors that affect population health.^118^ Interestingly, when Mendelian randomisation was performed researchers did not find any evidence supporting a causal relationship between genetically predicted serum vitamin D concentrations and the risk of RA,^119^ and more research is needed.

### Dietary and other factors

Nutrition, in general, has been shown to affect auto-immunity strongly.^120^ In regards to RA, studies have indicated that the Mediterranean diet, which relies on the daily consumption of extra virgin olive oil, fish, and omega-3 fatty acids, has a protective effect on RA development and clinical expression.^121–123^ On the other hand, high sodium, and red meat consumption, which are more typical in the Western diet, have been related to an increased risk of inflammatory polyarthritis or RA.^124,125^ A Western diet is also associated with an augmented risk of obesity, defined as abnormal fat accumulation in the human body and considered a significant overall health risk and, according to some studies, a possible risk for RA.^126^ Other eating habits, including fasting-mimicking diets, which have become increasingly popular, appear to benefit inflammation and RA disease activity. Yet, studies have not found any protective role of fasting on disease development.^127^

Moreover, studies indicate a possible relation between nutrition and gut microbiota as interconnected factors that impact RA risk. Dysbiosis, an imbalance in gut microbiota, is believed to be a contributing factor to RA that provokes an abnormal immune response and inflammation. A diet with high dietary fiber and reduced carbohydrate intake can improve gut microbiota composition in RA patients, while omega-3 fatty acids and reduced sodium intake can lower the risk of RA.^128^ Clinical trials have also shown that dietary interventions, including probiotics, can alleviate RA severity by reducing pathogenic bacteria and improving intestinal barrier and immune function.^128,129^

Finally, coffee consumption has been reported that may have a protective effect on the disease. Nevertheless, data from a Mendelian randomisation analysis do not support causal associations between coffee consumption and the development of RA.^130^

Regarding the use of medicines, there are data relating the use of statins with a lower risk of mortality among patients with RA.^131^ Statins indeed have pleiotropic effects ameliorating cardiovascular risk and inflammation in the context of RA.^132^ Interestingly some studies have shown that the risk of the disease may be lower in patients with higher versus lower statin treatment persistence or intensity. Still, future observational studies considering differences in dosage, duration of use, study population, and other factors should shed light on this observation.^133^

## CONCLUSIONS

Genetic and epigenetic factors, and environmental exposure, such as air pollution and lifestyle, influence the disease occurrence and clinical expression. Still, the multifactorial nature of RA makes studies of sole environmental factors challenging to interpret. As each factor may explain a small proportion of cases and cannot explain the disease’s complete underlying aetiology, the epidemiological studies’ results can be problematic. Stratifying patients according to their genetic profile and then identifying the environmental triggers for each subgroup may be more efficient. The ultimate goal should be to use nutritional and environmental modifiable risk factors in a prevention strategy that might help limit the consequences of the disease.

## ABBREVIATIONS

ACPA: anti-citrullinated protein antibodies;
ACR: American College of Rheumatology;
CS: cigarette smoking;
CRP: C-reactive protein;
CTLA4: Cytotoxic T-Lymphocyte Associated Protein 4;
DMARDs: disease-modifying antirheumatic drugs;
EBV: Epstein-Barr virus;
ESR: erythrocyte sedimentation rate;
FSH: follicle-stimulating hormone;
GWAS: Genome-wide association studies;
HLA: human leukocyte antigen;
IL: Interleukin;
miRNAs: microRNAs;
OCP: Oral contraceptives;
PADI: peptidylarginine deiminases;
P. gingivalis: Porphyromonas gingivalis;
PTM: Post-translational modifications;
RA: Rheumatoid arthritis;
RF: rheumatoid factor;
SE: shared epitope;
SMRs: standardized mortality ratios;
SNPs: single nucleotide polymorphisms;
PTPN22: tyrosine phosphatase non-receptor type 22;
STAT4: signal Transducer and Activator of Transcription 4;
TNF-α: tumour necrosis factor alpha.
